# Attention-deficit/hyperactivity disorder in Mozambique: an epidemiological investigation in a primary school sample

**DOI:** 10.47626/1516-4446-2023-3343

**Published:** 2024-03-25

**Authors:** Helena Mutede Cutótua Daniel, Igor Duarte, Arthur Caye, Antonio Suleman, Wilza Fumo, Luis Augusto Rohde

**Affiliations:** 1 Departamento de Psiquiatria e Saúde Mental, Hospital Psiquiátrico de Nampula, Nampula, Moçambique; 2 Programa de Pós-Graduação em Psiquiatria, Universidade Federal do Rio Grande do Sul (UFRGS), Porto Alegre, RS, Brazil; 3 Instituto Nacional de Psiquiatria do Desenvolvimento & Centro Nacional de Pesquisa e Inovação em Saúde Mental, São Paulo, SP, Brazil; 4 Programa de Déficit de Atenção e Hiperatividade, Hospital de Clínicas de Porto Alegre, UFRGS, Porto Alegre, RS, Brazil; 5 Departamento de Saúde Mental, Ministério da Saúde, Maputo, Moçambique; 6 Conselho Médico, Grupo UniEduK, São Paulo, SP, Brazil

**Keywords:** Attention-deficit/hyperactivity disorder, inattention, hyperactivity, prevalence, Africa

## Abstract

**Objective:**

To evaluate the prevalence of attention-deficit/hyperactivity disorder (ADHD), comorbidity rates with disruptive behavior disorders and main negative outcomes in primary school students in Nampula, Mozambique.

**Methods:**

We selected a random sample of 748 students for ADHD screening from a population of around 43,000 primary school students. The Swanson, Nolan, and Pelham Rating Scale version IV was applied to both parents and teachers. All students who screened positive (n=76) and a propensity score-matched random subset of students who screened negative (n=76) were assessed by a child psychiatrist.

**Results:**

The prevalence of ADHD was estimated at 13.4% (95%CI 11.5-19.2), and 30.6% of those with ADHD presented comorbid disruptive behavior disorders. Students with ADHD (n=36) had significantly higher rates of both substance use (alcohol, marijuana) (p < 0.001), and school failures than controls (n=96; p < 0.001). Comorbidity between ADHD and disruptive behavior disorders increased the chance of substance use (p < 0.001). Secondary analyses with more restrictive ADHD diagnostic criteria revealed a lower prevalence rate (6.7%; 95%CI 5.2-12.9) with similar patterns of associated factors and negative outcomes.

**Conclusion:**

Our findings demonstrated that ADHD is a prevalent mental disorder in Mozambique, and it is associated with similar comorbid profiles, predisposing factors, and negative outcomes, as in other cultures.

## Introduction

Attention-deficit/hyperactivity disorder (ADHD) is a neurodevelopmental disorder characterized by impaired and age-inappropriate levels of inattention and hyperactivity/impulsivity, manifesting early in development and influencing personal, social, or academic/professional functioning.^1^ ADHD has been associated with negative outcomes such as poor school performance and social rejection. Children with ADHD are more likely than their peers to develop conduct disorder in adolescence and antisocial personality disorder in adulthood, thereby increasing the likelihood of substance use disorders and arrests. Individuals with ADHD are also more likely to have injuries, accidents, and traffic violations, and may have an increased likelihood of obesity, sleep-onset latency, autoimmune diseases, sexually transmitted infections, metabolic disorders, pregnancy, and sexually transmitted diseases in adolescence. As a result, individuals with ADHD have a higher mortality rate than the general population.^1^

Meta-analytical data suggest a worldwide prevalence rate of ADHD in children and adolescents of around 5.3%.^2^ However, a subsequent meta-analysis indicated that the rate was even higher (7.2%).^3^ However, there was high heterogeneity among the studies in these meta-analyses, mainly due to methodological factors, such as different diagnostic approaches, information sources, and reported impairment.^2^ The most recent meta-analyses reviewing previous Global Burden of Disease data on ADHD found that the prevalence in children/adolescents was twice as high (5.41%, 95%CI 4.67-6.15) as in studies up to 2013 based on the same data source (2.68%, 95%CI 1.83-3.72%), with no significant differences between low-, middle-, and high-income countries.^4^ Recent data from specific countries, including the United States, have suggested an even higher prevalence rate of the disorder. In a telephone survey of 76,000 parents, 13% of school-age boys in the United States were diagnosed with ADHD, compared to 6% of school-aged girls. Among young people aged 12 to 17 years, the ADHD prevalence rate was even higher: 13.2%.^5^

Few studies have been conducted on ADHD in Africa. A meta-analysis of ADHD studies in sub-Saharan Africa reported prevalence rates ranging from 5.4 to 8.7% among children.^6^ In the same direction, an even more recent systematic review of African investigations^3^ suggested that the prevalence rate of ADHD was around 7.47% in children and adolescents. A wide variation in ADHD prevalence was observed across countries, ranging from 1.49% in Ethiopia to 11% in Uganda. However, it is important to highlight the difficulties obtaining adequate data from the studies in the two African reviews due to methodological issues, such as information collected from a single source, restricted age ranges, prevalence rates determined from screening instruments alone, no investigation of comorbid mental disorders, and small sample sizes. Moreover, there are no epidemiological data on ADHD in several African countries, including Mozambique. The results of the previous systematic review and meta-analysis in Africa also suggested that: 1) the prevalence of ADHD was considerably higher in males than females, with a male:female ratio of 2.01:1; and 2) the predominantly inattentive type (ADHD-I) is the most common ADHD presentation. These findings are similar to studies from other continents.^3^,^7^

There is extensive literature showing that, besides a well-established genetic risk for ADHD,^8^ several psychosocial factors are associated with ADHD, such as very/extreme prematurity, low birth weight, maternal age, hypertensive disorders during pregnancy, maternal preeclampsia during pregnancy, maternal obesity, low social class, paternal criminality, and maternal mental disorder.^1^ However, few studies have evaluated ADHD-associated factors in African countries. Their findings suggest that ADHD is associated with maternal substance use before and during pregnancy, a history of maternal abnormal vaginal discharge during pregnancy, perinatal complications, and low family education and/or sociodemographic status.^7^,^9^ Thus, one very relevant research question is whether we should expect higher prevalence rates in Africa since a higher prevalence of psychosocial risk factors is the rule in developing countries in the continent.^7^

The clinical relevance of studying ADHD in African children lies in its possible association with worse adaptive and social functioning, reflected both by higher rates of drug abuse/dependence and school failures and suspensions, which increase the probability of school dropout.^3^ Again, few studies have documented these negative outcomes in African countries. Some studies have reported that affected children are more likely to sustain a physical injury or cause injury to others, to have home accidents like hot water burns, drowning, or accidental ingestion of harmful substances, in addition to poor academic performance.^10^

Several studies have shown a high rate of comorbidity in children and adolescents with ADHD.^9^,^11^ Disruptive behavior disorders (DBD), i.e., conduct disorder and oppositional defiant disorder, are the main comorbidities in children and adolescents. ADHD prevalence studies in adolescents and cohort studies following children diagnosed with ADHD until adolescence, based on clinical and community samples, found joint occurrence of ADHD and conduct disorder or oppositional defiant disorder in about 30 to 50% of the cases.^12^,^13^ The association between ADHD in adolescence and drug abuse or dependence is also high.^14^ However, doubts persist whether associations between ADHD and both low school performance and drug abuse/dependence are not moderated by this frequent comorbidity between ADHD and conduct disorder.^11^,^15^

Thus, the main aim of this study was to evaluate the prevalence rate of ADHD using DSM-5 criteria in a primary school sample of Mozambican children. Secondary goals were: 1) to determine whether ADHD in youths from Mozambique is correlated with similar associated psychosocial factors in the international literature; and 2) to investigate the association between ADHD and negative outcomes such as school failure and drug use. Based on the literature, we hypothesized that: 1) the prevalence rate of ADHD and associated psychosocial factors in primary school students from the capital of the most populous province of Mozambique (Nampula)^16^ will be similar to those found in other African countries; 2) ADHD will be associated with more school failures; 3) students with ADHD will present significantly more early substance use than their peers without ADHD; and 4) the association between ADHD and both school failures and drug use is moderated by comorbid DBD.

## Methods

Our sample included primary school students (age range: 6 to 18 years) from 106 schools in Nampula City. The population of Nampula, the capital city of Nampula Province in northern Mozambique, is 743,125.^16^ It is the third largest city in Mozambique.^16^

### Sampling procedures

The sample size for the prevalence study was calculated using data provided by the Nampula Provincial Directorate of Education and Human Development on the number of primary school students enrolled in private and public schools in Nampula City for the 2019 school year. The total number of students in the 106 schools (private: 23 [830 students]; public: 83 [41,729 students]) was estimated at 42,559. An alpha error of 5%, measurement precision of 2%, an ADHD prevalence of 7.47%, and a sample loss of 10% were used in the calculation. This estimated prevalence was based on a recent African meta-analysis of ADHD^8^ among children and adolescents. The minimum sample size was estimated at 748 students.

Simple random sampling was carried out considering all the students enrolled in municipal primary schools (n = 42,559 students), both public (n=83) and private (n=23). The schools were numbered from 1 to 106 in alphabetical order. The students from the first school were numbered in alphabetical order from 1 to the maximum number of students, beginning with the first-year class of primary school until grade 7. The first student in the second school received the number immediately following the last student in the first school. The process followed among all the schools until the last student received a number.

The inclusion criteria were: 1) children enrolled between the first and seventh grades in metropolitan Nampula, and 2) children aged between 6 and 17 years. The only exclusion criterion was the unavailability of a guardian and a teacher at the school to provide information or respond to questionnaires.

### Screening procedures

Once the randomly selected individual was identified in the school, a research assistant (medical student) contacted the child's parents through the guidance of teachers or by mobile phone. The research assistants helped both parents and teachers fill out the screening instrument. This interview lasted an average of 10 minutes and took place in a private environment in the school. If a student had dropped out of school or the parents were unable to come to the school, the instrument was completed at the student’s home. If the class had more than one teacher, preference was given to the teacher who best knew the child at school (class tutor, the teacher with the greatest number of periods with the student, or, if it was impossible to define, the Portuguese or Mathematics teacher).

We used the validated Portuguese version of the Swanson, Nolan, and Pelham Rating Scale version IV as a screening instrument.^17^ This scale has been frequently used in ADHD investigations, including those designed to assess epidemiology and clinical interventions.^17^ The scale’s internal consistency varies from good to excellent.^17^ Its 26 items correspond to the DSM-IV criteria for ADHD and oppositional defiant disorder symptoms. Parents assess inattentive (items 1-9), hyperactive-impulsive (items 10-18), and defiant (items 19-26) behaviors using a 4-point Likert scale ranging from 0 (not at all) to 3 (too much).

Sudents with six or more inattentive and/or hyperactivity/impulsivity symptoms occuring at least frequently according to parent and/or teacher reports were considered positive cases. Although a lower cutoff point has already been validated in a previous epidemiological study in Brazil (five symptoms),^18^ we needed to increase the cutoff point for logistic reasons (i.e., to avoid an unmanageable number of diagnostic assessments). All positive cases were evaluated in the diagnostic confirmation phase. A sample was taken from propensity scores among non-cases, using age and biological sex as pairing variables. Thus, for each case identified in each school, there was a control from the same school of the same age and sex.

### Diagnostic procedures

All diagnostic assessments were performed by the first author (HMCD), who is a child and adolescent psychiatrist in Mozambique with extensive training in ADHD diagnosis at the Programa de Déficit de Atenção e Hiperatividade, Hospital de Clínicas de Porto Alegre, Brazil. She was blinded to the screening results. Separate interviews with youths and parents were conducted at the Psychiatric Hospital of Nampula or at the student’s school or home for those that could not attend the appointment at the hospital. Written consent was again obtained from all parents and adolescents older than 12 years at this stage.

DSM-5 criteria were used for diagnostic assessment of ADHD and DBD. The interviewer applied the ADHD and DBD modules of the Kiddie Schedule for Affective Disorders and Schizophrenia for School-aged Children. Clinical assessment was performed to assess major depressive disorder, any psychotic syndrome, and autism spectrum disorder. Only a clinical suspicion of intellectual disability was noted, since we could not conduct neuropsychological tests for intelligence quotient.

In addition to the psychiatric evaluation, the interviewer completed the Children’s Global Assessment Scale^19^ with the parents, as well as the items included in the Attention Problem subscale of the Child Behavior Checklist – parent report form.^20^ The Children’s Global Assessment Scale is a widely used measure of global functioning in children and adolescents with adequate psychometric properties (test-retest and inter-rater reliability/concurrent and discriminative validity).^19^ Its scores range from 0 to 100: the higher the score, the better the global functioning.^21^ The Attention Problems scale of the Child Behavior Checklist has high discriminatory power for clinical diagnosis of ADHD.^22^ All scales and instruments had been previously translated to Portuguese.

To satisfy the diagnostic criteria for ADHD, the child or adolescent had to completely fulfill all DSM-5 criteria (A, B, C, D, and E) in the clinical evaluation with the parents and the student. This approach has been widely used in other epidemiological studies.^23^ The student also had to score < 80 on the Children’s Global Assessment Scale (reflecting at least mild impairment) and have symptoms reported in two places (i.e., school report cards, church/mosque, or friend’s house, besides home) to be considered an ADHD case. This was to ensure that DSM-5 ADHD criteria C and D (pervasiveness and functional impairment) were fulfilled.^24^ It is important to note that the clinician conducting the assessment did not use teacher reports from the Swanson, Nolan, and Pelham Rating Scale version IV from the previous screening phase of the study. This decision was made because the reliability of teacher reports in Mozambique is extremely variable, considering that class sizes are enormous for Western standards (average 50-90 students), which increases the chance of false negatives. We estimated prevalence rates using an AND rule (symptoms threshold achieved by parents’ reports and teachers reporting at least three symptoms in at least one dimension) in secondary analyses.

Information about sociodemographics, substance use, school failures, and suspensions and expulsions was systematically collected from parents, teachers, and students. Substance use was assessed as alcohol, tobacco, marijuana, or inhalant use in the previous month and the total number of alcohol intoxication episodes in the students’ lives using Alcohol, Smoking, and Substance Involvement Screening Test,^25^ which was developed with the support of the World Health Organization. Its purpose is to screen cases of problematic use of psychoactive substances, mainly by primary health care professionals. This test, which has been translated into several languages and validated in Brazil,^25^ is a screening questionnaire consisting of eight questions. Information on pregnancy, maternal age at birth, prematurity, and birth weight was extracted from the student’s birth certificate, complemented by a report from the mother or a relative.

### Data analysis

The ADHD prevalence was estimated according to the following formula: P_e_ = [CDD + NSN (1 – NPV)]/n, where P_e_ = estimated prevalence, CDD = cases detected in the diagnostic phase, NSN = the number of screening negatives who were not evaluated in the diagnostic phase, NPV = negative predictive value of the screening test, and n = sample size. The CI for the estimated ADHD prevalence was calculated using the Fleiss quadratic approximation,^26^ and considering only the 132 clinically assessed students as the total observations (n), rather than the 722 students evaluated by the screening instrument. Using this approach, the CI was wider but avoided reflecting the uncertainty of interviewing a subsample of 132 students and then extrapolating from that to the rest of the sample.

All categorical variables were compared using the chi-square test. Since the vast majority of continuous data were not normally distributed, non-parametric tests (the Mann-Whitney *U* test) were used in this analysis. The Child Behavior Checklist’s Attention Problem sub-scale scores were compared between ADHD cases and non-cases using raw data.^20^ We used logistic regression analyses to compare binary outcomes and independent variables adjusted for confounders. The statistical significance level was set at 5%. All analyses were conducted in R software.

### Ethics statement

This study was approved by the Hospital de Clínicas de Porto Alegre Research Ethics Committee and the National Bioethics Committee for Health in Mozambique. Written consent was obtained from all parents and teachers. All children gave verbal consent, and adolescents aged between 12 and 17 years also provided written informed consent.

## Results

### Sample and symptomatic presentation in the screening phase

Of the randomly selected sample of 748 children and adolescents, screening could not be performed for 26 (3.5%) for the following reasons: i) refusal to participate (15), ii) death (one), and iii) school dropout (10). Thus, the final sample consisted of 722 adolescents. The median age was 10.3 years (range: 5-18 years); 45.4% (n=328) were boys and all students were between the first and the seventh grades.

According to the screening data, the most frequent inattention symptom reported by parents was “Seems not to be listening when directly spoken to” (13%), and the least frequent was “Is often forgetful during daily activities” (7.7%). In the teacher’s assessment, the symptom “Has difficulty organizing tasks and activities” (15%) was the most frequent, and “Distracted by external stimuli” (8.7%) was the least frequent. The most frequent symptom of hyperactivity/impulsivity reported by parents was “Excessive talking” (14.8%) and the least frequent was “Difficulty playing or engaging in leisure activities calmly” (8.6%). In the teacher’s assessment, the symptom “Interrupts others or intrudes (in conversations, games, etc.)” (15.1%) was the most frequent, while the least frequent was “Moves his hands or feet or wiggles in his chair” (5.8%). Using the same data, 76 (10.5%) adolescents reached or exceeded our threshold of six symptoms of inattention or hyperactivity/impulsivity either by parent or teacher report.

### Prevalence (primary objective)

For each student who screened positive, we randomly selected a control (who screened negative), using propensity scores adjusted by age and biological sex (76 students). We assessed 132 eligible students (86.8%), because 14 refused to participate in the diagnostic phase of the study, and six moved away and could not be found. Thirty-six students were considered cases with ADHD in the diagnostic phase (30 who screened positive and six who screened negative). In the remaining 96 non-ADHD controls, 42 screened positive and 54 screened negative.

The ADHD prevalence was estimated at 13.4% (95%CI 11.5-19.2). Among the 36 ADHD cases, nine (25%) were of the combined type, 14 (38.9%) were predominantly ADHD-I, and 13 (36.1%) were predominantly hyperactive/impulsive type.

### Demographic data, comorbidity, and associated factors

Comparisons were made between children and adolescents with (n=36) and without (n=96) a DSM-5 diagnosis of ADHD. The demographic characteristics of ADHD cases and non-cases are described in Table 1. There were significant differences between groups for age (the ADHD group was older; U = 640.5; p < 0.001) and biological sex (more boys in the ADHD group; χ^2^ = 7.85; degrees of freedom [df] = 1; p < 0.001).

Comorbid DBD occurred in 11 ADHD cases (30.6%; conduct disorder = 2.8%; oppositional defiant disorder = 30.6%). In the clinical assessment, we found no ADHD case with comorbid depressive disorder or psychosis. However, there was a clinical suspicion of intellectual disability in eight (22.2%) students with ADHD, and three (8.3%) had comorbid autism spectrum disorder. This was higher than the prevalence of these disorders in students without ADHD (χ^2^ = 9.73; df = 1; p < 0.001). The only variables significantly associated with ADHD in our sample were prematurity (cases = 13.8%; controls = 1%; χ^2^ =7.22; df = 1; p < 0.001) and infant medical problems at birth (cases = 8.3%; controls = 0%; χ^2^ = 4.86; df = 1; p = 0.027). However, we found no significant association between ADHD and: i) whom the child lived with; ii) maternal use of other medications besides vitamins and iron, and/or alcohol/drugs during pregnancy; iii) maternal smoking during pregnancy; iv) maternal illness or accident during pregnancy; v) birth weight; vi) family history of mental disorders (all p > 0.05) (Table 2).

In agreement with parental reports, the cases had a tendency, although not statistically significant, toward higher scores than non-cases on the Attention Problems scale of the Teacher Rating Form (U = 1,366.5; p = 0.06). The proportion of students with ADHD who had at least one school failure (32 out of 36; 88.9%) was significantly higher than that of non-cases (15 out of 96, or 15.6%) (χ^2^ = 61.29; df = 1; p < 0.001). No suspensions or expulsions were reported in our sample.

There were significant differences between students with and without ADHD regarding substance use (alcohol, tobacco, marijuana, inhalants) according to parental report (χ^2^ = 7.39; df = 1; p < 0.001). The prevalence of substance use was 11.1% in the ADHD group and 1% in the non-ADHD group.

To determine whether the association between ADHD and school failure was influenced by comorbid DBD, we ran logistic regression analyses using ADHD and DBD as the main factors and age and biological sex as covariables. The results indicated that both ADHD and DBD were associated with school failure (respectively z = 4.67, p < 0.001 and z = 2.24, p = 0.025). Moreover, the interaction between the two disorders significantly increased the chance of school failure (z = -2.43, p = 0.014). As expected, age (z = 3.546, p < 0.001) was also a significant predictor of failure.

To determine whether the association between ADHD and substance use was influenced by comorbid DBD, we also ran logistic regression analyses using ADHD and DBD as the main factors and age and biological sex as covariables. None of the variables were associated with substance use disorders in logistic regression, possibly due to the low prevalence of substance use disorders in the non-ADHD group.

### Secondary analyses

As a sensitivity analysis, we considered students to be ADHD-positive when they fulfilled the same definition used above but also had at least three inattentive and/or hyperactive/impulsive symptoms according to teachers in the screening assessment. This conservative definition was used to increase the probability that some ADHD symptoms occurred at school. The ADHD prevalence was estimated at 6.7% (95%CI 5.2-12.9). Among the 25 identified ADHD cases, seven (28%) were the combined type, nine (36%) were predominantly ADHD-I, and nine (36%) were the predominantly hyperactive/impulsive type. According to this definition, the same pattern of findings emerged in comparisons between cases and controls, except biological sex and medical conditions at birth. No significant association was detected between this more conservative definition of ADHD and these two variables.

## Discussion

In a sample of Mozambican students, we found an ADHD prevalence of 13.37%, high comorbidity between ADHD and DBD (30.6%), as well as significant associations between ADHD and prematurity, medical conditions at birth, and school failures. Comorbidity with DBD increased the risk of school failure. ADHD was associated with substance use. To the best of our knowledge, this is the first epidemiological study on ADHD in Mozambican children and adolescents.

The ADHD prevalence rate found in this study (13.4%) was higher than that detected in worldwide meta-analyses,^2^,^4^,^27^ and both a meta-analysis and a systematic review from Africa.^3^,^6^ Potential reasons for this are: 1) studies using DSM-5 criteria tend to show higher ADHD prevalence rates than those using DSM-IV criteria because the former considers the age of onset to be 12 years, whereas the DSM-5 describes an earlier age of onset (6 years). In addition, the DSM-5 does not exclude diagnosis in the presence of autism spectrum disorder^28^; 2) our ADHD case definition included mild impairment (Children’s Global Assessment Scale < 80). Many studies have required more severe impairment, and previous meta-analyses have shown that the definition of impairment impacts prevalence rates^2^; 3) our prevalence was based on parental report only. When teacher-reported symptoms were also considered, the prevalence fell within international rates.

The male/female ratio of ADHD cases in our sample was approximately 2:1 (66.7/33.3%). A similar ratio was found in other African studies^3^,^6^ and a worldwide meta-analysis.^2^ Recently, an investigation reassessing ADHD findings using 2019 Global Burden of Disease data found an incidence rate 2.5 to 2.6 times higher among males than females.^4^ However, the high prevalence of DSM-5 DBD in this sample of young Mozambicans with ADHD is in agreement with several studies that demonstrated high comorbidity between ADHD and DBD.^2^,^15^,^18^

Surprisingly, we found no comorbidity between ADHD and depression, although it is suggested in the literature.^29^ Some investigations propose that the construct of depression in African cultures might be different than that of instruments developed in Western cultures.^30^ As expected, ADHD cases had a higher prevalence rate of both intellectual disability and autism spectrum disorder than students without ADHD. However, clinical suspicion of intellectual disability was more prevalent in our sample was higher than that of African children,^31^ which probably reflects screening differences. Regarding autism spectrum disorder, our prevalence of comorbid ADHD was similar to that of previous studies.^32^

Our findings suggest an association between ADHD and both prematurity and a medical condition at birth. Similar findings have occurred in previous investigations worldwide.^33^,^34^ In contrast, we found no association between ADHD and several other potential risk factors. Considering the most discussed risk factors in the literature (birth weight, smoking during pregnancy, and family history of mental disorders), it is important to note that: 1) although not statically significant, our ADHD group had a lower birth weight (median = 2,765 g) than the non-ADHD group (median = 2,945g). Thus, we might not have had sufficient power to detect the difference; 2) previous studies have suggested that the association between smoking during pregnancy and ADHD is confounded by genetic risk^35^; 3) our assessment of family mental disorders was not structured and was only based on a general question for the parents.

We found significant associations between ADHD and substance use and school failure in this sample. Moreover, comorbid DBD increased the risk of school failure. These results partially replicated the findings of a previous investigation in Brazil.^18^ Other studies worldwide have already demonstrated these associations.

As expected, we found a lower ADHD prevalence rate when we required several teacher-reported symptoms in our definition of the disorder. Epidemiological ADHD studies using the “and rule” (both parental and teacher reports) instead of the “or rule” clearly document the same.^2^ Since our *a priori* definition of ADHD was based only on parent and child clinical assessment, we used findings based on parental and teacher report in secondary analysis. In addition, previous investigations have suggested that teachers might not detect the inattentive presentation of ADHD (the most prevalent type in our and other epidemiological studies) and part of our sample was from grades in which students had many teachers, decreasing awareness of student ADHD symptoms.^18^ Moreover, primary school classes in Mozambique have many more students than those in high- and middle-income countries (e.g., several had > 50 students per class). Thus, teacher ability to detect ADHD was likely reduced.

Some study limitations must be pointed out. Because we relied on a school setting to estimate the prevalence of ADHD, our findings must be generalized cautiously to the general population of children and adolescents. However, the demographic characteristics of the youths in this study (age range distribution and male/female ratio in these age ranges) are similar to those of the most recent national census (2017), except for the fact that number of students aged 15 to 18 years was lower than in the census, probably due to school dropouts.^16^ We did not formally asses intelligence quotient, using only a clinical suspicion of intellectual disability. Few 5-year-old children were assessed (a lower age than was stipulated in our inclusion criteria) since they entered the first grade at this age and turned 6 during the academic year. It was surprising that no mother reported alcohol/drug use or smoking during pregnancy or mental disorders among biological relatives. This might reflect recall bias, discomfort in disclosing drug use during pregnancy or a lack of awareness and mental health services in Mozambique. Finally, the sample size of ADHD students was relatively small, limiting our statistical power for examining the association between this diagnosis and certain factors and outcomes. However, most of our results are in agreement with recent reports with larger samples.

Although our ADHD prevalence rates were higher than previous investigations in Western and African countries, the pattern of comorbidities and impairments observed in this sample of students with ADHD from Mozambique is consistent with other reports worldwide.^18^,^36^,^37^ Thus, our findings support the cross-cultural validity of the DSM-5’s conceptualization of ADHD. Also, the significantly higher rates of school failures and substance use disorders in children and adolescents with ADHD in our sample reinforce the disorder’s negative impact in different cultures.

Despite the above mentioned caveats, we conclude that ADHD is a highly prevalent disorder among Mozambican children and adolescents, and it is associated with clinically significant functional impairment. Large-scale prospective studies on ADHD in Mozambique and other African countries are needed to further explore the impact of the clinical and epidemiological features of this disorder during development, increasing our knowledge on ADHD in low- and middle-income countries.

## Disclosure

AC provided one consultancy session for Knight Therapeutics in 2022. LAR has received grant or research support from, served as a consultant to, and served on the speakers’ bureau of Abdiibrahim, Adium, Apsen, Abbott, Aché, Bial, Medice, Novartis/Sandoz, Pfizer/Upjohn, and Shire/Takeda in the last 3 years; and has received authorship royalties from Oxford Press and ArtMed. The ADHD and juvenile bipolar disorder outpatient programs chaired by LAR have received unrestricted educational and research support from the following pharmaceutical companies in the last 3 years: Novartis/Sandoz and Shire/Takeda. The other authors report no conflicts of interest.

## Figures and Tables

**Table 1:** Demographic data and school failures in students with and without ADHD

Characteristics	ADHD (n=36)	Non-ADHD (n=96)	p-value
Age (years)			
5-10	8 (22.2)	61 (63.5)	
11-15	18 (50.0)	33 (34.4)	< 0.01
16-18	10 (27.8)	2 (2.1)	
Sex			
Male	24 (66.7)	36 (37.5)	< 0.01
Female	12 (33.3)	60 (62.5)	
Ethnicity			
Black	36 (100.0)	96 (100.0)	NS
School failure			
Yes	32 (88.9)	15 (15.6)	< 0.01
No	4 (11.1)	81 (84.3)	

Data presented as n (%), unless otherwise specified.

ADHD = attention-deficit/hyperactivity disorder; NS = non-significant.

**Table 2:** Factors assessed between students with and without attention-deficit/hyperactivity disorder.

Variable	Non-ADHD (n=96)	ADHD (n=36)	p-value†
With whom is the student currently living?			0.31
Both biological parents	74 (77.1)	30(83.3)	
Biological mother and stepfather	9 (9.4)	1 (2.8)	
Biological father and stepmother	5 (5.2)	0 (0.0)	
Biological mother only	2 (2.1)	1 (2.8)	
Biological father only	0 (0.0)	1 (2.8)	
Grandfather/grandmother	5 (5.2)	3 (8.3)	
Other relative/friend	1 (1.0)	0 (0.0)	
Did the mother use other medicines besides vitamins and iron?	0 (0.0)	0 (0.0)	-
Did the mother use alcohol/drugs during pregnancy?	0 (0.0)	0 (0.0)	-
Did the mother smoke during pregnancy?	0 (0.0)	0 (0.0)	-
History of mental disorders among biological relatives	0 (0.0)	0 (0.0)	-
Prematurity	1 (1.0)	5 (13.9)	0.01
Infant medical problems at birth	0 (0.0)	3 (8.3)	0.02

Data presented as n (%), unless otherwise specified.

† Fisher's exact test.
